# Special Issue: Advanced Science and Technology of Polymer Matrix Nanomaterials

**DOI:** 10.3390/ma16165551

**Published:** 2023-08-09

**Authors:** Peijiang Liu, Liguo Xu, Jinlei Li, Jianping Peng, Zhenkai Huang, Jintang Zhou

**Affiliations:** 1 Reliability Physics and Application Technology of Electronic Component Key Laboratory, The Fifth Electronics Research Institute of the Ministry of Information Industry, Guangzhou 510610, China; cz2343222@163.com; 2 College of Light Chemical Industry and Materials Engineering, Shunde Polytechnic, Foshan 528333, China; 3 Science and Technology on Space Physics Laboratory, Beijing 100076, China; 4 School of Materials Science and Hydrogen Energy, Foshan University, Foshan 528000, China; pjp7@outlook.com (J.P.); hzk@fosu.edu.cn (Z.H.); 5 College of Materials Science and Technology, Nanjing University of Aeronautics and Astronautics, Nanjing 211100, China

Polymer matrix nanomaterials have revolutionized materials science due to their unique properties resulting from the incorporation of nanoscale fillers into polymer matrices [1,2,3]. These nanofillers, which include nanoparticles, nanotubes, and nanosheets, offer a high surface-to-volume ratio and exceptional mechanical, thermal, and electrical characteristics [4]. As a result, polymer matrix nanocomposites exhibit enhanced performance compared to their pristine polymer counterparts. The ability to tailor the properties of polymer matrix nanomaterials by controlling the size, shape, and distribution of nanofillers has sparked considerable interest in both academia and industry.

The synthesis and fabrication of polymer matrix nanomaterials plays a crucial role in determining their properties and performance. To achieve well-dispersed and homogeneous nanofiller distribution within the polymer matrix, innovative processing techniques such as in situ polymerization, melt blending, electrospinning, and layer-by-layer assembly have been developed [5,6,7]. These methods enable precise control over the dispersion and orientation of nanofillers, improving the interfacial interactions and overall material performance. In addition, the exceptional properties of polymer matrix nanomaterials have led to their widespread applications in various fields. In the realm of electronics, nanocomposites have been employed for flexible and stretchable electronics, high-performance printed circuit boards, and advanced packaging materials [8,9]. The integration of nanofillers in polymer matrices has also revolutionized energy storage devices, enabling the development of high-capacity batteries, supercapacitors, and fuel cells [10,11]. Moreover, the unique properties of polymer matrix nanomaterials have been harnessed to fabricate sensors with enhanced sensitivity, selectivity, and stability [12,13]. In the biomedical field, nanocomposites have shown great potential for drug delivery systems, tissue engineering scaffolds, and biosensors, owing to their biocompatibility and tunable properties [14,15,16].

Polymer matrix nanomaterials represent a cutting-edge area of research with immense potential for various applications. For this Special Issue, “Advanced Science and Technology of Polymer Matrix Nanomaterials”, we are seeking contributions in the form of original research articles and reviews in the field of polymer matrix nanomaterials, highlighting their synthesis, characterization, and applications. This Special Issue aims to explore the wide-ranging applications of polymer matrix nanomaterials, including (but not limited to) electronics, energy storage, sensors, and biomedical devices. Continued research efforts and collaborations across disciplines will drive the advancement and utilization of polymer matrix nanomaterials, leading to scientific and technological breakthroughs.
